# Integrative Multi-Omics Analysis Identifies IL18R1 as a Circulating Prognostic Biomarker for Risk Stratification in Extensive-Stage Small Cell Lung Cancer

**DOI:** 10.3390/cancers18101608

**Published:** 2026-05-15

**Authors:** Shengjuan Hu, Sicong Li, Yiyuan Cui, Ying Wang, Luyao Chen, Xiyuan Zhang, Li Hou, Li Feng

**Affiliations:** 1 Department of Hematology and Oncology, Dongzhimen Hospital, Beijing University of Chinese Medicine, Beijing 100700, China; hsj18810165527@163.com; 2 Traditional Chinese Medicine Department, National Cancer Center/National Clinical Research Center for Cancer/Cancer Hospital, Chinese Academy of Medical Sciences and Peking Union Medical College, Beijing 100021, China; lisicong32@163.com (S.L.); cuiyiyuan369@126.com (Y.C.); xiyuan1117@163.com (X.Z.); 3 Department of Oncology, The Mongolia-Chinese Medical Hospital of Chifeng, Chifeng 024000, China; wangying990901@163.com; 4 The First Affiliated Hospital of Guangdong Pharmaceutical University, Guangzhou 511436, China; cly32318356098974@163.com; 5 Beijing Key Laboratory of Digital Intelligence Traditional Chinese Medicine Oncology Prevention and Treatment, Beijing 100021, China

**Keywords:** biomarker, IL18R1, machine learning, single-cell analysis, small cell lung cancer

## Abstract

**Background**: Small cell lung cancer (SCLC) carries a dismal prognosis with limited biomarkers for risk stratification. This study aimed to identify circulating prognostic biomarkers. **Methods**: We prioritized SCLC risk-associated genes using Summary-data-based Mendelian Randomization of pQTL/eQTL, differential expression, and weighted gene co-expression network analysis. Five machine learning approaches were compared to develop a diagnostic model based on *ACE*, *AGER*, and *IL18R1*, trained on GSE149507 and validated in GSE60052. We conducted single-cell transcriptomic analysis using public datasets (GSE150766 and GSE279570) and peripheral blood mononuclear cells (PBMCs) from our extensive-stage cohort. Finally, prioritizing the lead candidate IL18R1, we enrolled a prospective clinical cohort to assess its prognostic utility. A LASSO–Cox prognostic model incorporating plasma IL18R1 and clinical variables was internally validated (7:3 split) for progression-free survival (PFS) prediction. **Results**: Integrative multi-omics identified *ACE*, *AGER*, and *IL18R1* as SCLC-protective genes. Elastic Net machine learning identified a two-gene predictive signature (*AGER* and *IL18R1*) with robust diagnostic accuracy. Single-cell RNA sequencing revealed the predominant downregulation of *ACE*, *AGER*, and *IL18R1* in T cells and alveolar type II cells from SCLC patients. PBMC analysis further supported *IL18R1* downregulation in CD8^+^ T cells, NK cells, and dendritic cells. In an independent prospective cohort (*n* = 300), lower plasma IL18R1 levels were independently associated with shorter PFS (HR = 0.997 per unit increase; 95% CI: 0.995–0.999; and *p* = 0.003), with time-dependent AUCs of 0.77–0.86. Performance in limited-stage disease was inconsistent and requires further validation. A prognostic model incorporating plasma IL18R1 and 11 clinical parameters stratified patients into distinct risk groups (HR = 5.19), showing a strong discriminative ability in extensive-stage SCLC. **Conclusions**: We identified *ACE*, *AGER*, and *IL18R1* as protective factors against SCLC progression. Integration of plasma IL18R1 with clinical parameters provides a prognostic tool for extensive-stage SCLC.

## Simple Summary

Small cell lung cancer is a highly aggressive disease with poor survival outcomes. Currently, doctors lack reliable tools to predict which patients will fare worse, making personalized treatment difficult. This study sought to identify new blood-based markers for risk stratification. Through comprehensive genetic analysis, we discovered candidate protective genes and focused on *IL18R1*. We found that patients with lower plasma IL18R1 experienced faster cancer progression. By combining plasma IL18R1 measurements with routine clinical information, we developed a risk prediction model that effectively distinguishes high-risk from low-risk patients, particularly in extensive-stage small cell lung cancer. This approach holds potential for enhancing risk stratification, which may help identify vulnerable patients earlier and inform more tailored treatment strategies.

## 1. Introduction

Small cell lung cancer (SCLC) accounts for 10–15% of lung cancers, with an aggressive course and 5-year survival under 7% [1]. Despite frontline immunotherapy for extensive-stage (ES) SCLC, most patients relapse during maintenance, and monotherapy benefits only 10–15% of cases [2,3]. While *TP53* and *RB1* alterations are nearly universal [4], they lack actionable targets. Emerging therapies include antibody–drug conjugates, DNA damage response inhibitors, and CAR T cells [5,6], yet clinical management remains limited by poor efficacy, rapid resistance, and the absence of predictive biomarkers.

We conducted a multi-omics study to identify and validate novel SCLC molecular targets through three integrated stages. First, we combined Summary-data-based Mendelian Randomization (SMR), differential expression analysis, and weighted gene co-expression network analysis (WGCNA) to identify causal hub genes, followed by a machine learning-based diagnostic evaluation [7]. Second, single-cell RNA sequencing characterized gene expression patterns in the tumor microenvironment, with findings explored in an exploratory pilot analysis of peripheral blood mononuclear cells (PBMCs). Third, we developed a prognostic model incorporating the top candidate (plasma IL18R1) with routine clinical parameters to predict progression-free survival (PFS) and enable risk stratification.

## 2. Materials and Methods

### 2.1. Summary-Data-Based Mendelian Randomization

#### 2.1.1. SCLC Data Acquisition and Quantitative Trait Locus Data Sources

SCLC Genome-Wide Association Study (GWAS) summary statistics were obtained from FinnGen (179 cases/218,613 European ancestry controls) [8]. Transcriptome datasets (GSE149507, GSE60052) were retrieved from Gene Expression Omnibus (GEO). Blood expression quantitative trait loci (eQTL) data were derived from eQTLGen (*n* = 31,684) [9] and cis-protein quantitative trait loci (cis-pQTLs) from a meta-analysis of five GWASs (734 proteins, r^2^ < 0.001; *p* < 5 × 10^−8^, excluding MHC region) [10,11,12,13,14].

#### 2.1.2. SMR Analysis, Protein–Protein Interaction Network Construction, and Bayesian Colocalization Analysis

SMR with heterogeneity in dependent instruments (HEIDI) testing was performed to integrate GWAS data with eQTL and pQTL data [7,15], utilizing SMR software (v1.3.1). The HEIDI test distinguished pleiotropy from linkage disequilibrium. Associations with FDR < 0.05 and HEIDI *p* > 0.05 were deemed significant. For protein–protein interaction (PPI) network analysis, genes passing FDR < 0.002 were analyzed using STRING (v11.5) and visualized in Cytoscape (v3.9.1); hub genes were identified by degree centrality. To complement the SMR analysis and assess whether the genetic associations with gene expression and SCLC risk share the same causal variant, we performed Bayesian colocalization using the coloc R package (v5.2.2). For each candidate gene identified in SMR, we extracted cis-eQTL summary statistics (±1 Mb from the transcription start site) and the corresponding SCLC GWAS summary statistics from FinnGen. We computed the posterior probability of five mutually exclusive hypotheses: H0 (no association with either trait), H1 (association with gene expression only), H2 (association with SCLC only), H3 (association with both traits, distinct causal variants), and H4 (association with both traits, sharing the same causal variant). A posterior probability of H4 (PP.H4) > 0.75 was considered strong evidence for colocalization, 0.5–0.75 as moderate evidence, and <0.5 as weak or no evidence.

### 2.2. Differential Expression Analysis, WGCNA, and Functional Enrichment

Differential expression analysis of GSE149507 was performed using the “limma” R package (v3.54.4). Differentially expressed genes (DEGs) were defined as genes with |log_2_ fold change| > 1 and adjusted *p* < 0.05 (Benjamini–Hochberg). WGCNA was conducted to construct co-expression networks. A soft-thresholding power of β = 18 (R^2^ > 0.9) was selected to ensure scale-free topology. Modules were identified using the dynamic tree-cut algorithm (merge cut height = 0.25) and correlated with clinical traits (Pearson). Hub genes were identified based on gene significance (GS) and module membership (MM), with thresholds set at |GS| > 0.2 and |MM| > 0.8. Functional enrichment analysis was performed using the “clusterProfiler” R package (v4.12.1). Candidate genes from SMR, WGCNA, and DEG analyses were intersected using Venny 2.1.0.

### 2.3. Machine Learning Model Construction and Evaluation

Candidate gene classification was evaluated using five algorithms: ElasticNet (glmnet v4.1-8), Support Vector Machine (e1071 v1.7-14), eXtreme Gradient Boosting (xgboost v1.7.7.1), k-Nearest Neighbors (FNN v1.1.4; caret v6.0-94; parsnip v1.2.0), and Random Forest (randomForest v4.7-1.1). Models were trained on the GSE149507 dataset (*n* = 36) and externally validated using GSE60052 (*n* = 86). Discrimination was assessed by the area under the curve (AUC), Matthews correlation coefficient, sensitivity, specificity, Negative Predictive Value, Positive Predictive Value, and F1-score, with the Youden index determining optimal cutoffs. Calibration was evaluated using the Brier score and Hosmer–Lemeshow test, and variable importance was analyzed via SHAP values for the best-performing algorithm.

To prevent information leakage and optimism bias, we implemented a 5 × 5 nested cross-validation (NCV) framework for all machine learning models. The outer loop (5-fold stratified cross-validation) provided unbiased performance estimates, while the inner loop (5-fold cross-validation) performed hyperparameter tuning strictly within the training folds of each outer iteration. Anti-leakage safeguards included the following: (i) hyperparameter selection (e.g., XGBoost max_depth/eta/nrounds, Elastic Net λ, SVM cost/gamma, KNN k, and Random Forest mtry) was performed exclusively via inner CV; (ii) standardization parameters (mean/SD for SVM and KNN) were computed on training folds and applied to test folds; (iii) the optimal classification threshold was determined by the Youden index on each outer test fold; and (iv) no feature pre-screening or model selection was conducted on the full dataset prior to NCV. Permutation testing (100 iterations) was performed for each model by randomly shuffling outcome labels while preserving feature structure. Each permuted dataset underwent identical NCV, and empirical *p*-values were calculated as the proportion of permuted AUCs greater than or equal to the observed NCV AUCs. The predefined independent validation cohort was retained for external generalizability assessment but was not used for model training, hyperparameter optimization, or threshold determination.

### 2.4. Single-Cell Analysis

#### 2.4.1. Sample Collection and Data Acquisition

We collected peripheral blood from 3 treatment-naïve ES-SCLC patients and 3 healthy controls under institutional ethics committee (Approval No. 23/511-4254) with written informed consent. Public scRNA-seq datasets were retrieved from GEO: GSE150766 (SCLC tumor, *n* = 1) and GSE279570 (lung tissues: 7 healthy controls and 11 COPD patients). Normal lung epithelial cells from GSE279570 healthy donors were utilized as reference controls for comparative analyses with SCLC cells from GSE150766.

#### 2.4.2. Library Preparation and Sequencing

Single-cell suspensions were processed on the 10x Genomics Chromium platform. Reverse transcription and cDNA amplification utilized the Chromium Next GEM Single Cell 3′ Kit (10x Genomics, Pleasanton, CA, USA). Libraries were quality-assessed using an Agilent Bioanalyzer 2100 (Agilent Technologies, Santa Clara, CA, USA) and sequenced on an Illumina NovaSeq 6000 (Illumina, San Diego, CA, USA) (150 bp paired-end reads, ~50,000 reads/cell) (150 bp paired-end reads, ~50,000 reads/cell). Raw data were analyzed with Cell Ranger (v6.0) for alignment to GRCh38, barcode processing, and generation of gene–cell count matrices.

#### 2.4.3. Quality Control and Preprocessing of Single-Cell Dataset

Downstream analyses were performed using the Seurat R package (v4.4.0). Stringent quality control metrics were implemented to retain cells meeting the following criteria: (i) detection in ≥3 cells; (ii) gene counts between 200 and 7500; and (iii) mitochondrial content < 10%. Normalization and variance stabilization were conducted using either SCTransform or conventional log-normalization followed by scaling. Inter-dataset batch effects were mitigated using Harmony or integration anchors (Seurat CCA). The top 2000 variable genes were chosen by vst. Following principal component analysis, graph-based clustering (resolution = 0.2) produced 14 clusters, annotated via Human Primary Cell Atlas and SingleR (v2.4.1).

#### 2.4.4. Differential Expression, Intercellular Communication, and Pathway Enrichment Analysis

Comparative transcriptomic analysis employed FindAllMarkers (min.pct = 0.25, log_2_FC = 0.25), with DEGs illustrated using DotPlot and FeaturePlot. Cell–cell communication patterns were deciphered using CellChat (v2.1.1) to detect ligand–receptor interactions. Gene Set Enrichment Analysis (GSEA) was executed with clusterProfiler against Kyoto Encyclopedia of Genes and Genomes pathways (c2.cp.kegg.v2023.2.Hs), with enrichment significance set at |NES| > 1 and FDR < 0.25.

### 2.5. Clinical Validation of Plasma IL18R1 as a Prognostic Biomarker in SCLC

#### 2.5.1. Study Design and Participants

This prospective cohort study recruited patients with newly diagnosed, treatment-naïve SCLC at the Cancer Hospital, Chinese Academy of Medical Sciences (Beijing, China), between February 2022 and November 2025. Inclusion criteria comprised: (i) pathologically confirmed SCLC (histologically or cytologically) based on WHO 2021 classification; (ii) receipt of stage-appropriate first-line therapy per institutional protocols and Chinese Society of Clinical Oncology guidelines—specifically, limited-stage (LS) SCLC patients underwent concurrent chemoradiotherapy (cisplatin 75 mg/m^2^ day 1 plus etoposide 100 mg/m^2^ days 1–3 for 4 cycles combined with thoracic radiotherapy 45–60 Gy/25–30 fractions starting day 1 of cycle 1 or 2), followed by prophylactic cranial irradiation or hippocampal-avoidant prophylactic cranial irradiation for responders; ES-SCLC patients received 4–6 cycles of carboplatin (AUC 5–6 day 1) and etoposide (100 mg/m^2^ days 1–3) combined with atezolizumab (1200 mg IV every 3 weeks), followed by maintenance immunotherapy until disease progression, unacceptable toxicity, or withdrawal; (iii) Eastern Cooperative Oncology Group performance status ≤ 2; (iv) age ≥ 18 years; (v) adequate hematologic, renal, and hepatic function (hemoglobin ≥ 90 g/L, absolute neutrophil count ≥ 1.5 × 10^9^/L, platelet count ≥ 100 × 10^9^/L, total bilirubin ≤ 1.5 × upper limit of normal [ULN], serum creatinine ≤ 1.5× ULN, and alanine/aspartate aminotransferase [ALT/AST] ≤ 2.5 × ULN); and (vi) documented informed consent.

Subjects were excluded for (i) previous SCLC-directed systemic therapy; (ii) history of other cancers within 5 years; (iii) autoimmune disorders requiring systemic immunosuppression; (iv) active or chronic infections (e.g., HIV, active hepatitis B/C); (v) pregnancy or lactation; or (vi) inability to adhere to study procedures.

Pre-treatment blood samples were collected within 72 h before first-line therapy for plasma IL18R1 quantification.

#### 2.5.2. Sample Size Calculation

Sample size was estimated for PFS validation, assuming HR = 0.85 per SD increase in the composite risk score (derived from the multivariable prediction model incorporating plasma IL18R1 and clinicopathological variables), α = 0.05, 80% power, median PFS = 6 months, and 1:1 allocation. This required 260 evaluable patients (~130 events), with a 15% dropout allowance yielding a target enrollment of 300. Calculations used used R (v4.5.3) with the powerSurvEpi package (v0.1.3).

#### 2.5.3. Study Endpoints, Follow-Up, and Censoring Criteria

PFS was defined as the interval from frontline therapy initiation to first documented progression (Response Evaluation Criteria in Solid Tumors [RECIST] 1.1) or death. Radiological assessments were performed every 6–9 weeks during the first year and every 12 weeks thereafter. Censoring and discontinuation criteria were predefined as:

(i) Withdrawal of consent: Voluntary patient request to discontinue study participation. These patients (*n* = 12) were excluded from both primary and sensitivity analyses and did not contribute to the complete-follow-up cohort (*n* = 300), in accordance with ethical requirements prohibiting data use after consent withdrawal.
(ii) Loss to follow-up: Failure to attend scheduled assessments for ≥3 months without documented clinical outcome. These patients were censored at last contact in the primary analysis but excluded from sensitivity analyses (*n* = 18).
(iii) Treatment discontinuation: Patients who discontinued first-line therapy prematurely (defined as receiving <4 cycles of chemotherapy, early cessation due to adverse events, or switch to non-protocol therapy). While retained in the primary analysis with continued follow-up, these patients (*n* = 14) were excluded from sensitivity analyses to assess model robustness.

For patients who remained progression-free at the data cutoff date (30 November 2025), survival time was censored at the cutoff date.

#### 2.5.4. Data Collection and Biomarker Assessment

Baseline data collection encompassed 29 clinical metrics prior to therapy, comprising patient demographics (age, sex, and body mass index [BMI]), hepatobiliary function markers (albumin, ALT, and AST), renal function (serum creatinine), comorbidity profiles (diabetes, coronary heart disease, hypertension, and hyperlipidemia), hematological indices (red blood cells [RBCs], hemoglobin, platelets, white blood cells [WBCs], neutrophils, and lymphocytes), oncological markers (pro-gastrin-releasing peptide [ProGRP], CA125, neuron-specific enolase [NSE], carcinoembryonic antigen [CEA], cytokeratin-19 fragment [CYFRA21-1], and squamous cell carcinoma antigen [SCC]), coagulation markers (thrombin time [TT], prothrombin time [PT], activated partial thromboplastin time [APTT], D-dimer, and fibrinogen), and plasma IL18R1 levels.

#### 2.5.5. Measurement of Plasma Soluble IL18R1 by ELISA

Plasma IL18R1 was quantified by ELISA (Human IL18R1 ELISA Kit; FineTest, Wuhan, China; catalog No. EH0155; and detection range: 31.25–1000 pg/mL) using EDTA-plasma samples. Blood was centrifuged at 4 °C within 30 min of collection; plasma was aliquoted and stored at −80 °C until analysis. Prior to use, the lyophilized standard was reconstituted and serially diluted with sample diluent to generate an 8-point standard curve (1000, 500, 250, 125, 62.5, 31.25, 15.625, and 0 pg/mL). Biotin-conjugated detection antibody and HRP-conjugated streptavidin (SABC) working solutions were freshly prepared within 30 min of use by 1:100 dilution of the concentrated stocks. Samples were analyzed in duplicate according to the manufacturer’s protocol. Briefly, 100 μL of standards or plasma samples were added to capture antibody-coated wells and incubated at 37 °C for 90 min, followed by two washes without soaking. Biotin–antibody working solution (100 μL) was then added and incubated at 37 °C for 60 min, followed by three washes (1 min soak per wash). HRP-SABC working solution (100 μL) was subsequently added and incubated at 37 °C for 30 min, followed by five washes (1 min soak per wash). TMB substrate (90 μL) was added and incubated at 37 °C for 10–20 min, and the reaction was terminated with 50 μL of stop solution. Optical density was immediately read at 450 nm. Values exceeding the standard curve range (1000 pg/mL) were diluted and re-assayed. Intra-assay and inter-assay coefficients of variation were 4.2–4.84% and 4.13–4.16%, respectively.

#### 2.5.6. Statistical Analysis and Prognostic Model Construction

Data were randomly divided (7:3, stratified by stage) into training (*n* = 209) and validation (*n* = 91) sets. The overall case-wise missing data rate was 4.0% (12 of 300 patients). Missing data were imputed using MICE (mice v3.16.0). Least absolute shrinkage and selection operator (LASSO) regression identified candidate variables with non-zero coefficients (λ selected via 10-fold cross-validation), followed by multivariable Cox modeling. Continuous variables were Z-standardized during LASSO fitting (glmnet default) to ensure appropriate regularization; however, the final reported coefficients and hazard ratios are presented on the original scale (per unit increase). Proportional hazards assumption was verified (Schoenfeld test). A nomogram was developed using the rms package (v6.7-0) and internally calibrated via bootstrap (B = 1000). Traditional calibration plots were additionally generated for both the training and validation cohorts to graphically assess agreement between predicted and observed probabilities. Discrimination was assessed by time-dependent receiver operating characteristics analysis, with the optimal risk score cutoff determined using maxstat (maximally selected rank statistics with several *p*-value approximations version: 0.7-25). Patients were subsequently stratified into low- and high-risk groups for PFS comparison (Kaplan–Meier with log-rank test). Decision curve analysis (DCA; stdca v2.0) evaluated clinical utility. Stage-specific analyses were performed separately for LS- and ES-SCLC. The primary analysis was conducted on complete-follow-up cohort (*n* = 300). First-line complete cohort (*n* = 268) served as sensitivity analysis for the model performance. To evaluate the robustness of our findings to the missing data imputation strategy, we repeated the prognostic model development and validation using complete cases only (*n* = 288, after excluding patients with any missing values).

#### 2.5.7. Ethics Statement

This investigation adhered to the Declaration of Helsinki and received ethical approval from the review board of the Cancer Hospital, Chinese Academy of Medical Sciences (Approval No. 23/511-4254). All participants signed informed consent forms prior to study entry.

## 3. Results

**Figure 1:**
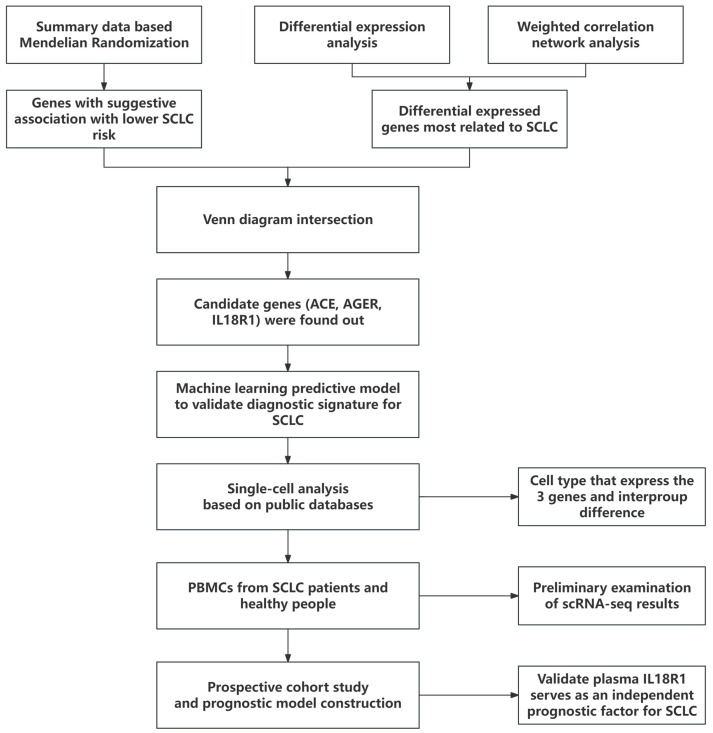
The study design.

The study flow is illustrated in Figure 1.

### 3.1. Identification of Three Suppressor Genes Through SMR, WGCNA, and DEG

**Figure 2:**
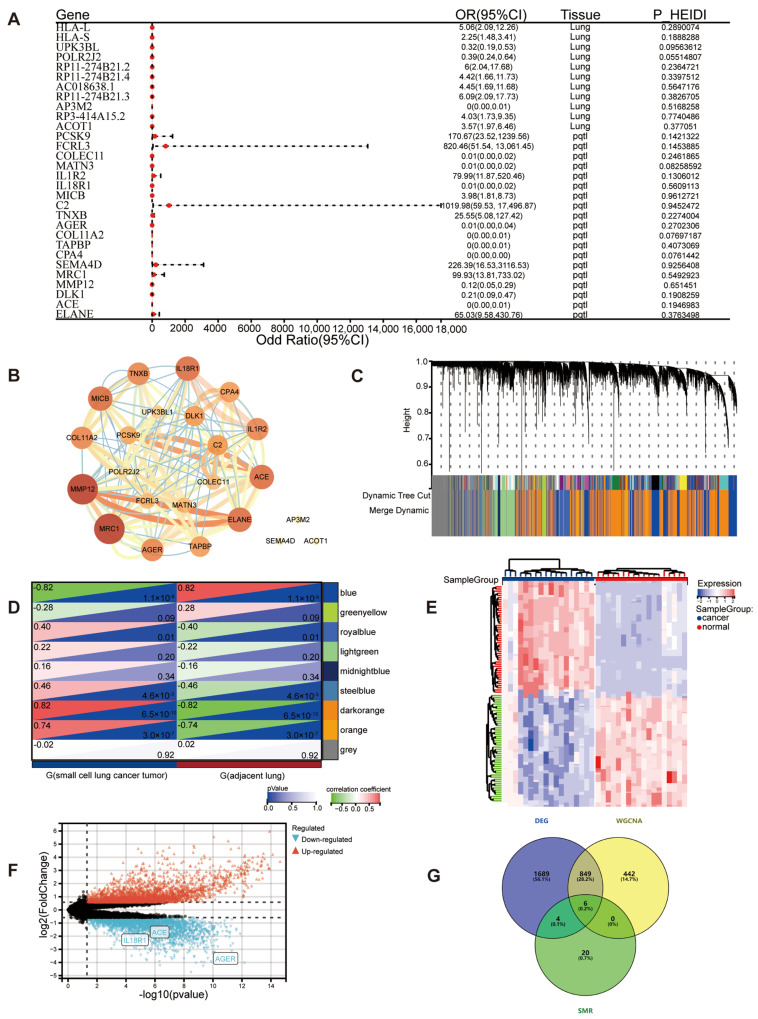
Identification of SCLC-related targets. (**A**) Forest plot depicting genes associated with small cell lung cancer. OR, odds ratio; CI, confidence interval. (**B**) Protein–protein interaction networks constructed from genes associated with small cell lung cancer. The seven genes *HLA-L*, *HLA-S*, *RP11-274B21.2*, *RP11-274B21.4*, *AC018638.1*, *RP11-274B21.3*, and *RP3-414A15.2* were not shown in the figure, since they were not included in STRING2. The color intensity of nodes corresponds to degree centrality, where darker colors denote higher degree values and lighter colors denote lower degree values. (**C**) Clustering dendrogram of WGCNA genes and module differentiation. Different colors represent different modules. (**D**) Module-trait heatmap of the correlation between co-expression modules and SCLC status in the GSE149507 dataset, with correlation coefficients indicated within each module. (**E**) Heatmap visualization of differentially expressed genes in GSE149507 (blue reflects downregulation, red reflects upregulation). (**F**) Volcano plot illustrating differential expression results for GSE149507. (**G**) Venn diagram revealed 6 overlapping genes from SMR, WGCNA, and DEG.

SMR analysis of 16,380,466 blood-based eQTL associations identified 11 eQTLs and 19 pQTLs significantly associated with SCLC (FDR-corrected *p* < 0.05, HEIDI *p* > 0.05) (Figure 2A). PPI networks were constructed for the resulting genes (Figure 2B). WGCNA of the GSE149507 dataset identified nine co-expression modules, with the blue module showing the strongest correlation with SCLC (Figure 2C,D; detailed module parameters and trait relationships in Figure S1A–C and Table S1). DEG analysis revealed 2419 upregulated and 2733 downregulated genes (Figure 2E,F and Table S2). The intersection of WGCNA and DEG results yielded 1236 overlapping candidate genes (Figure 2G), with functional enrichment indicating involvement in immune-related pathways (Figure S1D). Subsequent integration with SMR data identified six candidate genes (*ACE*, *AGER*, *PCSK9*, *MRC1*, *C2*, and *IL18R1*), of which three (*ACE*, *AGER*, and *IL18R1*) were prioritized based on protective effects (*b*_SMR < 0), module membership, and decreased tumor expression (log_2_FC < 0) as the final candidates (Figure 2G, Table S3).

To complement the SMR findings and assess whether the genetic associations with gene expression and SCLC risk share the same causal variant, Bayesian colocalization analysis was performed for the three genes with the strongest SMR signals. Moderate-to-strong evidence of a shared causal variant was observed for *AGER* (PP.H4 = 0.76), whereas *ACE* (PP.H4 = 0.21) and *IL18R1* (PP.H4 = 0.09) showed weak or no evidence of colocalization (Table S4, Figure S2).

### 3.2. Diagnostic Value of AGER and IL18R1 Revealed by Elastic Net Analysis

**Figure 3:**
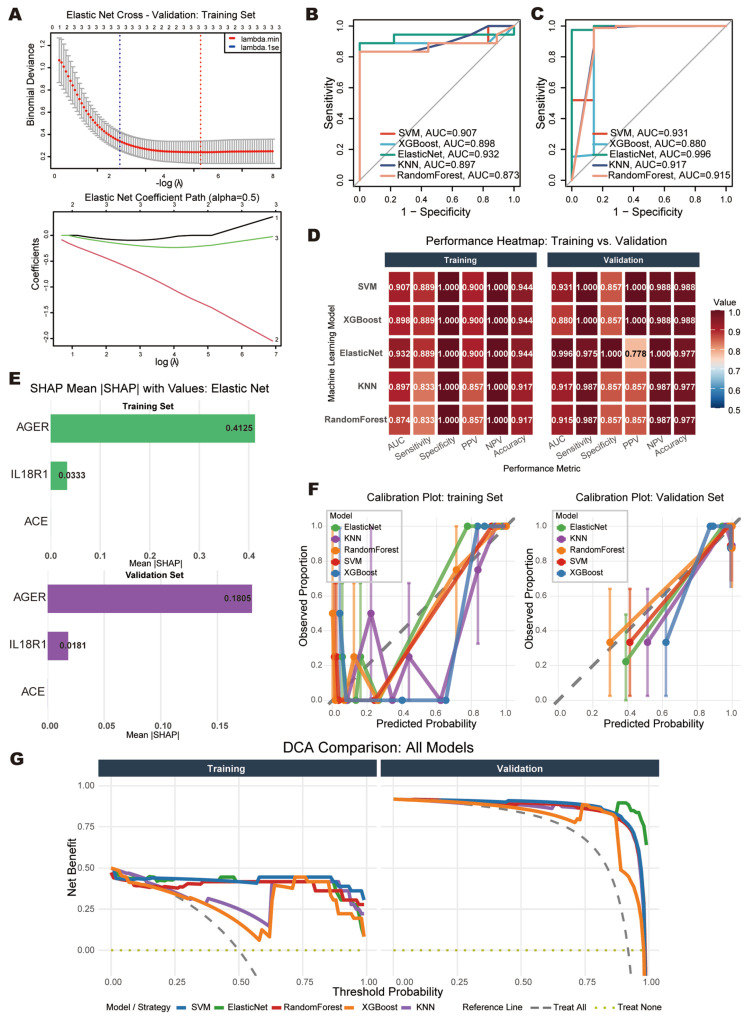
Machine learning to verify the diagnostic efficacy of candidate hub genes. (**A**) Elastic Net cross-validation (above) and coefficient paths (below). (**B**,**C**) Receiver operating curves comparing five machine learning algorithms in training (**B**) and validation (**C**) sets. (**D**) Performance comparison of machine learning models in the training and validation cohorts. (**E**) Mean |SHAP| values in training and validation sets. (**F**) Calibration curves showing the relationship between predicted probabilities and observed proportions. (**G**) Decision curve analysis (DCA) comparing the net clinical benefit of five machine learning models across threshold probabilities in the training and validation cohorts.

Five machine learning algorithms were compared for diagnostic model construction. Ten-fold cross-validation was performed exclusively on the training set to determine the optimal hyperparameter (λ = 0.022). The cross-validation (CV) coefficient path indicated three active variables at this λ value; however, refitting the final model on the complete training dataset with λ = 0.022 yielded non-zero coefficients for only two genes—*AGER* (β = −1.314310) and *IL18R1* (β = −0.210016)—with *ACE* being shrunk to exactly zero (β = 0.000000). This indicates that the higher expression of *AGER* and *IL18R1* is associated with lower SCLC risk (Figure 3A). Such a discrepancy between the CV path and the final model arises because the cross-validation curve aggregates sparsity patterns across multiple folds where variable selection may vary due to sampling variability, and when refitting on the complete dataset with the CV-determined λ, the coordinate descent algorithm may converge to a slightly different sparsity pattern. Consequently, *ACE* contributes no predictive value in the final model. The Elastic Net model demonstrated excellent discriminative ability in the validation cohort (AUC = 0.996, 95% CI: 0.988–1.000), significantly outperforming other models (Figure 3B,C). Notably, the validation set exhibited markedly different baseline expression profiles compared with the training set (*ACE*, *AGER*, and *IL18R1* all *p* < 0.001; Supplementary Table S5), suggesting that the discrimination reflects genuine population heterogeneity. Complete performance metrics are detailed in Supplementary Table S6, and the distribution of predicted probabilities for all models is illustrated in Supplementary Figure S3. A performance heatmap compared five machine learning algorithms across evaluation metrics in the training and validation sets (Figure 3D). SHAP analysis confirmed the contribution hierarchy in both the training and validation sets, with *AGER* exhibiting the highest mean |SHAP| value, followed by *IL18R1*, while *ACE* showed zero contribution. This confirms that *AGER* and *IL18R1* are the primary biological drivers of SCLC risk stratification, aligning with their differential expression patterns (Figure 3E). Traditional calibration plots revealed variable agreement between predicted and observed probabilities across the five machine learning models (Figure 3F). Decision curve analysis further demonstrated that ElasticNet achieved the highest net benefit across clinically relevant threshold probabilities (Figure 3G).

### 3.3. Cell Type-Specific Expression of ACE, AGER, and IL18R1 in T Cells and Alveolar Type II Cells Revealed by Single-Cell Analysis

**Figure 4:**
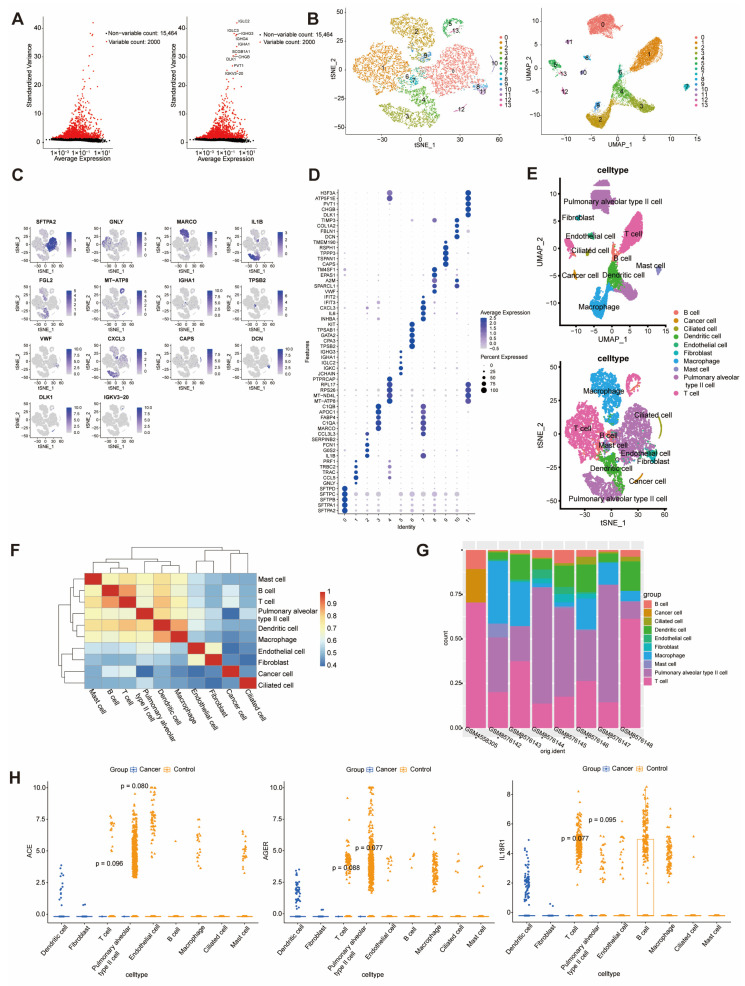
Single-cell analysis identified cell types and intergroup differences in each cell type in SCLC tissue. (**A**) Top 10 highly variable genes. (**B**) t-SNE and UMAP visualization resolving 14 cell clusters (colors indicate phenotypes). (**C**) Feature plots mapping marker gene distribution. (**D**) Dot plots of the top 5 markers per cluster. (**E**) Cell type annotation (10 populations: macrophages, alveolar type II cells, dendritic cells, T cells, mast cells, endothelial cells, ciliated cells, fibroblasts, cancer cells, and B cells). (**F**) Inter-cell type correlation heatmap. (**G**) Cluster proportions across samples. (**H**) *ACE*, *AGER*, and *IL18R1* expression by cell type.

Quality control of GSE150766 and GSE279570 yielded 772 and 14,731 genes, respectively. The top 10 highly variable genes are shown in Figure 4A. Dimensionality reduction (t-SNE/UMAP) identified 14 distinct cell clusters (Figure 4B), with the highest-expressed marker gene visualized across clusters (Figure 4C) and the top five markers per cluster illustrated in Figure 4D. Expression-based annotation identified 10 cell populations, including macrophages, dendritic cells, T cells, and cancer cells (Figure 4E), with correlation analysis revealing significant relationships between macrophage–dendritic and T-B cell lineages (Figure 4F). Cell type composition differed between SCLC and normal lungs, with B cells, T cells, and tumor cells enriched in SCLC (Figure 4G). Volcano plots highlight the top five DEGs (Figure S4). The differential expression of *ACE*, *AGER*, and *IL18R1* was primarily observed in T cells and alveolar type II cells (Figure 4H).

### 3.4. Cell Communication and Signaling Pathways Mediated by Candidate Genes

CellChat analysis revealed ligand–receptor interactions between dendritic cells/fibroblasts and cancer cells, involving key pairs including CD34–SELP, CD40LG–CD40, and CD274–PDCD1 (Figure S5). GSEA further indicated tumor-suppressive roles through specific signaling pathways: *ACE* upregulated the NOD-like receptor (NLR) and p53 signaling, *AGER* downregulated Hedgehog (Hh) signaling, and *IL18R1* activated p53 signaling in dendritic cells (Figures S6–S8).

### 3.5. Exploratory Single-Cell Analysis of Clinical PBMCs Suggests Differential Expression of IL18R1

**Figure 5:**
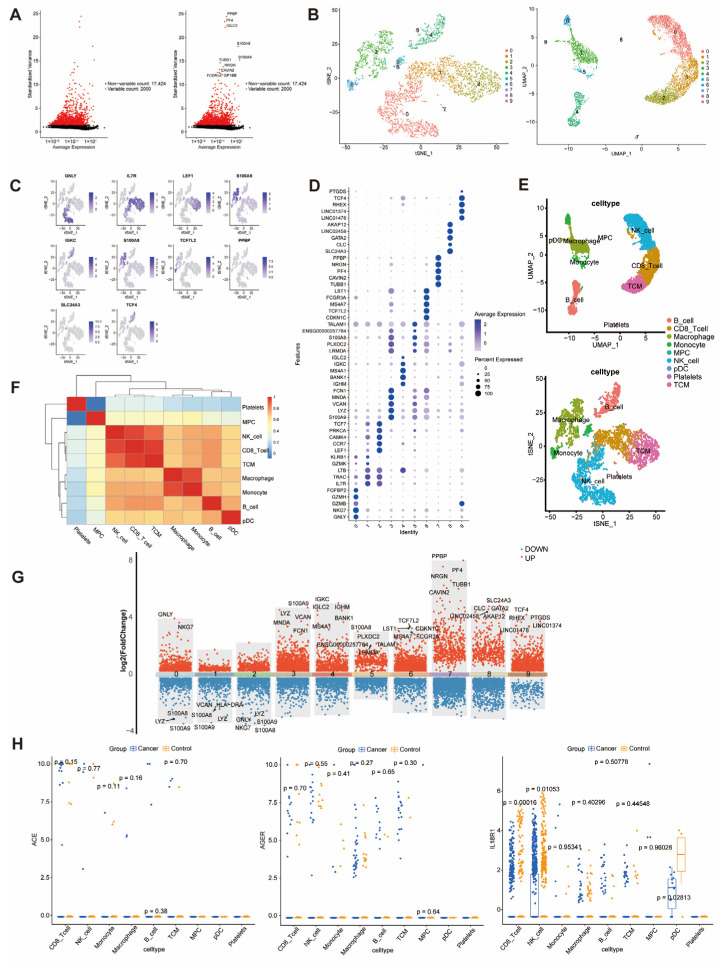
Single-cell analysis identified cell types and intergroup differences in each cell type in peripheral blood mononuclear cells. (**A**) Top 10 highly variable genes. (**B**) t-SNE and UMAP visualization of 9 cell clusters: B cell, CD8^+^ T cell, macrophage, Monocyte, mesenchymal progenitor cell (MPC), NK cell, plasmacytoid dendritic cell (pDC), platelets, and Central Memory T Cell (TCM). (**C**) Feature plots mapping marker distribution. (**D**) Bubble plots of top 5 markers per cluster. (**E**) Cell type classification. (**F**) Correlation heatmap. (**G**) DEG volcano plots (SCLC vs. healthy) with top 5 hits labeled. (**H**) *ACE*, *AGER*, and *IL18R1* expression by cell type.

After quality control and doublet removal, a total of 5965 cells and 19,424 genes were retained from three ES-SCLC patients and three healthy controls. Single-cell profiling of PBMCs identified nine distinct cell clusters (Figure 5A–E). Comparative analysis revealed significantly reduced *IL18R1* expression in CD8^+^ T cells, NK cells, and plasmacytoid dendritic cells (pDC) from SCLC patients versus healthy controls (*p* < 0.05), whereas *ACE* and *AGER* showed no significant differences (Figure 5F–H). These findings corroborate the tumor tissue data and implicate IL18R1 downregulation in systemic immune dysregulation in ES-SCLC. Given the limited sample size (*n* = 3 per group), this analysis was regarded as exploratory rather than definitive validation.

### 3.6. Plasma IL18R1 as an Independent Prognostic Indicator for ES-SCLC

**Figure 6:**
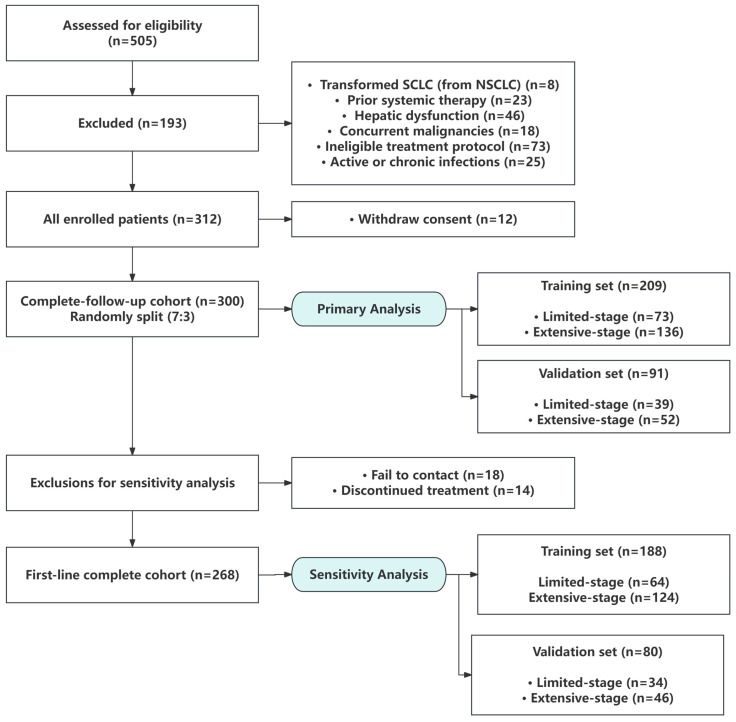
Study cohort diagram. Light blue shading distinguishes the analytical cohorts (primary analysis and sensitivity analysis) from the patient enrollment flow (white boxes).

**Figure 7:**
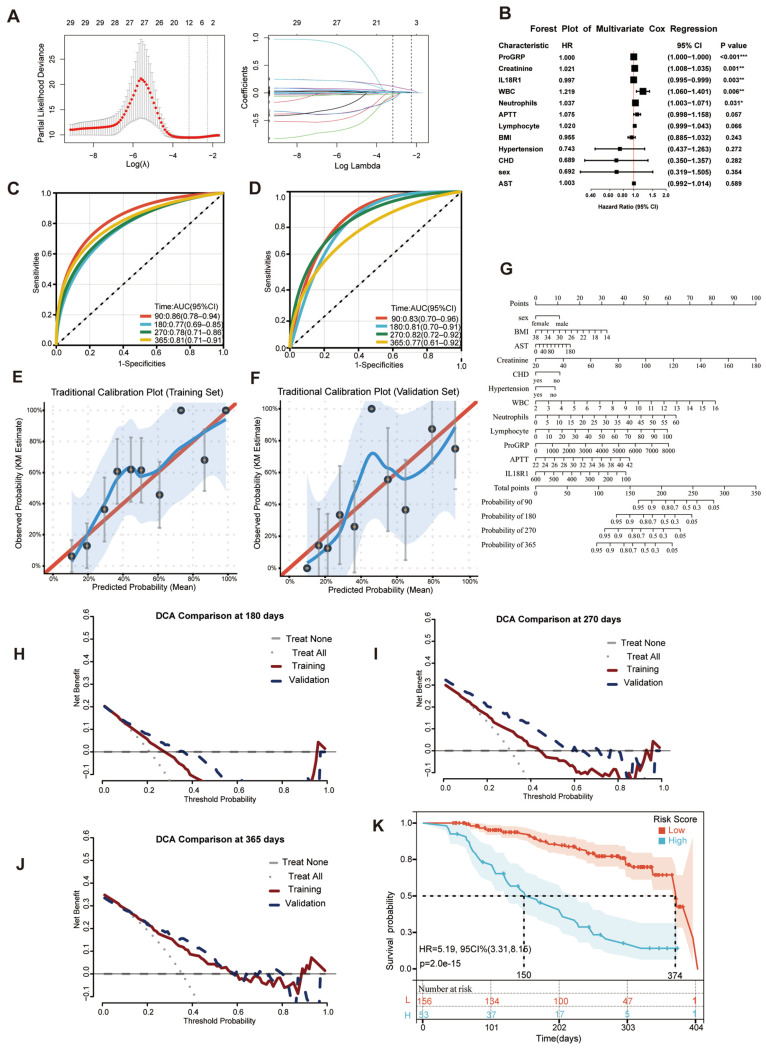
Construction and validation of the IL18R1-based prognostic model in complete-follow-up cohort. (**A**) LASSO coefficient paths for variable selection. Each line represents the coefficient path of one candidate variable; colors are for visual discrimination only. (**B**) Forest plot of multivariate Cox regression. CHD: coronary heart disease. * *p* < 0.05, ** *p* < 0.01, *** *p* < 0.001. (**C**,**D**) Time-dependent receiver operating characteristic curves for the training (**C**) and validation (**D**) sets of complete-follow-up cohort. (**E**,**F**) Traditional calibration plot of the IL18R1-based prognostic model in the training set (**E**) and validation set (**F**). The blue line represents the smoothed calibration curve; the light blue shaded area indicates its 95% confidence interval. The red line denotes the ideal perfect calibration (reference line). (**G**) Nomogram for predicting 3-, 6-, 9-, and 12-month PFS. (**H**–**J**) Decision curve analysis comparing net benefits. The model achieved maximum net benefits of 0.12 (training) and 0.13 (validation) at 180 days (**H**), 0.23 (training) and 0.26 (validation) at 270 days (**I**), and 0.28 (training) and 0.28 (validation) at 365 days (**J**), respectively. (**K**) Kaplan–Meier curves stratified by risk groups.

From 505 screened individuals, 312 (61.78%) were enrolled, and the primary analysis was conducted on the complete-follow-up cohort (*n* = 300), including 112 (37.33%) with LS and 188 (62.67%) with ES disease (Figure 6). No significant differences in baseline characteristics were found between the training and validation groups in either the complete-follow-up cohort or the first-line complete cohort (*p* > 0.05, Tables S7 and S8). Over a median follow-up of 13 months, the median PFS was 219 days (180-, 270-, and 365-day rates: 60.00%, 36.67%, and 10.00%). LASSO–Cox regression identified 12 variables from 29 candidates, with plasma IL18R1 emerging as a significant protective factor (HR = 0.997 per unit increase, 95% CI: 0.995–0.999; *p* = 0.003) (Figure 7A,B). The full set of 12 selected variables, including their coefficients, hazard ratios, and 95% confidence intervals, is presented in Supplementary Table S9. The Cox model demonstrated a moderate discriminative performance, with time-dependent AUCs ranging from 0.77 to 0.86 in the training set and 0.77–0.83 in the validation set across 90–365 days (Figure 7C,D). The C-index was 0.753 and 0.758, respectively. The calibration plots demonstrated good agreement between predicted and observed probabilities in both the training and validation cohorts, indicating a satisfactory calibration performance (Figure 7E,F). Detailed calibration metrics are provided in Supplementary Table S10. A nomogram was constructed for individualized PFS prediction (Figure 7G). DCA demonstrated favorable clinical utility, with the model outperforming “treat-none” strategies across clinically relevant thresholds (10–60%) and showing robust generalizability (Figure 7H–J). Patients were stratified into high- and low-risk groups based on the optimal cutoff identified by maxstat. Within the training set, median PFS was markedly prolonged in low-risk versus high-risk patients (374 vs. 150 days), with high-risk status conferring a 5.19-fold elevated hazard for progression (HR = 5.19, 95% CI: 3.31–8.15; *p* < 0.001) (Figure 7K).

**Figure 8:**
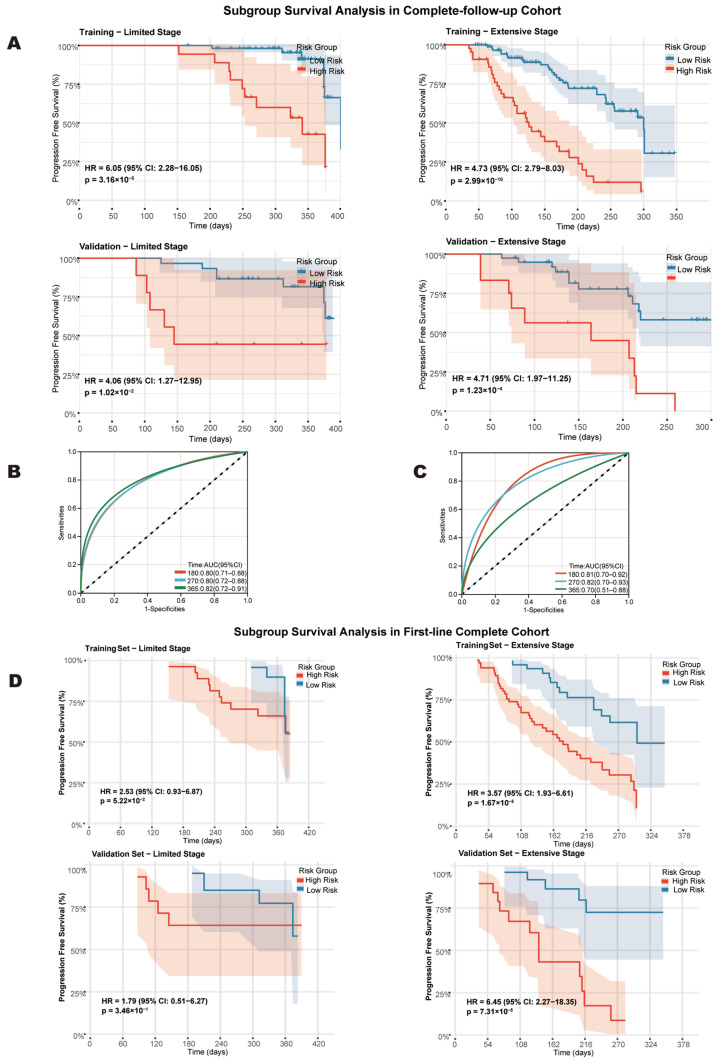
Subgroup analyses by disease stage and sensitivity validation. (**A**) Subgroup analysis by disease stage in complete-follow-up cohort. (**B**,**C**) Time-dependent Receiver Operating Character (ROC) curves for the training (**B**) and validation (**C**) sets of first-line complete cohort. (**D**) Subgroup analysis by disease stage in first-line complete cohort.

Subgroup analyses demonstrated consistent prognostic stratification across disease stages and datasets. In both LS (HR 6.05 [training] vs. 4.06 [validation]) and ES-SCLC (HR 4.73 vs. 4.71, all *p* < 0.001), high-risk status predicted a significantly shorter PFS, underscoring robust model generalizability (Figure 8A, Table S11). Sensitivity analyses in the first-line complete cohort (*n* = 268) confirmed consistent stratification for ES-SCLC, with high-risk patients showing a shorter median PFS (training: 178 vs. 301 days, HR = 3.57; validation: 139 days vs. not reached, HR = 6.45; and all *p* < 0.001) (Figure 8B,C). However, the prognostic performance was inconsistent for LS-SCLC, likely reflecting the limited sample size and low event rates in the validation cohort (Figure 8D).

To verify that our findings were independent of the MICE imputation approach, we performed an additional robustness check using complete cases only (*n* = 288). The results remained consistent with the primary analysis (Supplementary Figures S9 and S10 and Table S12), supporting the robustness of the model to missing data handling.

## 4. Discussion

SCLC remains a highly aggressive malignancy with dismal outcomes. We identified *ACE*, *AGER*, and *IL18R1* as putative tumor suppressors through integrative multi-omics analysis, with machine learning validating a predictive signature driven by *AGER* and *IL18R1*. Single-cell analysis further revealed distinct expression patterns within specific tumor microenvironment cell clusters. Clinical validation in patient-derived PBMCs specifically implicated *IL18R1*, prompting the focused evaluation of its prognostic utility. Our findings establish IL18R1 as a robust circulating biomarker for risk stratification in ES-SCLC, though validation in LS disease remains warranted.

*IL18R1* encodes the IL-18 receptor α-subunit, which mediates opposing effects through IL-18 (pro-inflammatory) or IL-37 (anti-inflammatory) signaling, depending on receptor complex composition [16,17,18]. The antineoplastic effects of IL-18 are primarily attributed to its capacity to facilitate maturation, activation, and the persistence of T cells within the tumor microenvironment. *IL18R1* deficiency accelerated tumor growth in the murine models of hepatocellular carcinoma by impairing the accumulation and effector function of tumor-infiltrating CD8^+^ and CD4^+^ T cells [19]. Rather than functioning as a canonical tumor suppressor gene, these observations indicate that *IL18R1* serves as a functional immune receptor that marks and sustains an inflamed, immune-active tumor microenvironment. In non-small cell lung cancer, IL18R1 is selectively expressed by the functional T-bet + Eomes + CD8^+^ T cell subset, distinguishing it from exhausted populations and correlating with robust IFN-γ production upon IL-18 stimulation [20]. Similarly, in lung squamous cell carcinoma, IL18R1 expression positively correlates with immune scores and the infiltration of cytotoxic cells, NK cells, and dendritic cells [21].

Notably, IL-37, an anti-inflammatory cytokine that attenuates immune responses, also signals through IL-18Rα (in complex with IL-1R8) to exert anti-neoplastic effects [16]. In lung squamous cell carcinoma, *IL18R1* overexpression suppresses cell proliferation, migration, and invasion, and its downregulation independently predicts poor prognosis [21]. To date, the role of *IL18R1* in SCLC remains undefined. Here, by analyzing publicly available scRNA-seq datasets, we demonstrate that *IL18R1* is significantly downregulated in SCLC tissues. In our patient cohort, IL18R1 was predominantly expressed by CD8^+^ T cells, NK cells, and plasmacytoid dendritic cells in PBMCs, suggesting that reduced IL18R1 may reflect a less inflamed or immune-excluded microenvironment. It is worth noting that, while membrane-bound IL18R1 is not secreted, its shed soluble form (sIL-18Rα) is the species detected in plasma [22,23]. Although plasma IL18R1 may reflect non-specific pulmonary inflammation in certain contexts [24,25], all patients in our cohort were free of active infection or overt inflammatory disease at the baseline. Thus, the prognostic signal observed here likely represents tumor-specific immune engagement via the IL-18/IL18R1 axis [26], consistent with an inflamed, immune-active microenvironment rather than non-specific pulmonary inflammation. IL18R1 downregulation in SCLC likely indicates diminished IL-18-mediated immune surveillance, which may contribute to immune evasion and disease progression.

*ACE*, predominantly expressed in pulmonary capillary endothelium, is markedly reduced or absent in SCLC and neuroendocrine carcinomas, likely reflecting tumor-induced vascular destruction [27,28]. Mendelian randomization studies further suggest that reduced ACE activity increases SCLC risk [29], supporting its protective function. *AGER*, expressed on pulmonary epithelial cells, suppresses NSCLC progression through cell cycle regulation [30,31,32], and its downregulation has been associated with increased lung cancer risk in genetic association studies [33]. In our study, both genes were significantly downregulated in SCLC tissues and exhibited protective associations in SMR analysis, consistent with their roles in maintaining pulmonary homeostasis. These findings align with our observation that *IL18R1*, similarly downregulated in SCLC immune cells, represents part of a broader transcriptional signature of compromised tumor suppression in the pulmonary microenvironment.

Regarding the predictive model, the Elastic Net classifier demonstrated moderate performance, successfully distinguishing SCLC patients from healthy controls. Single-cell analysis revealed that *ACE*, *AGER*, and *IL18R1* were predominantly expressed in dendritic cells, fibroblasts, T cells, and alveolar type II cells, with significant downregulation observed in T cells and alveolar type II cells. PBMCs analysis further validated *IL18R1* downregulation in CD8^+^ T cells, NK cells, and dendritic cells, supporting its potential as a minimally invasive biomarker.

GSEA suggests these genes suppress SCLC through distinct immunomodulatory mechanisms. *IL18R1* and *ACE* were associated with p53 pathway upregulation in dendritic cells—which is critical given that TP53 is inactivated in ~95% of SCLC cases [4]. Restoring p53 signaling may induce cell cycle arrest and enhance antigen presentation [34]. *ACE* further correlated with NLR signaling activation, potentially promoting inflammasome-dependent cytokine maturation and enhancing DC activation while suppressing regulatory T cells [35]. Conversely, *AGER* appeared to confer protection through Hh pathway inhibition. Hh hyperactivation in SCLC promotes cancer stem cell maintenance and immunosuppression [36,37,38,39]; thus, AGER-mediated suppression may restore the dendritic cell immunostimulatory capacity. The modulation of these genes held particular significance for SCLC, an immunologically “cold” tumor characterized by poor T cell infiltration. The concordance between tissue and PBMC expression patterns for *IL18R1* underscores its translational potential as an accessible prognostic indicator.

Therefore, we integrated plasma IL18R1 with routine clinical laboratory parameters using LASSO–Cox regression to develop a prognostic model for risk stratification. Given the rapid progression and high recurrence rates characteristic of SCLC, we selected PFS as the primary endpoint. IL18R1 remained statistically significant in multivariate analysis (HR = 0.997 per unit increase, *p* = 0.003), consistent with our bioinformatics findings. While the independent per-SD effect was modest, its integration into the prognostic model contributed to robust discrimination (time-dependent AUC > 0.75, C-index ~0.75) and clinically meaningful stratification: high-risk patients exhibited a substantially shorter median PFS (150 vs. 374 days) with a 5.19-fold increased progression risk. This marked divergence highlights the model’s potential to identify patients at high risk for rapid progression who may benefit from intensified monitoring or alternative therapeutic strategies.

The prognostic model maintained discriminative ability across both LS- and ES-SCLC in the complete-follow-up cohort. This suggests potential as a universal risk stratification tool independent of TNM classification, addressing the limited prognostic granularity of current staging systems. However, sensitivity analyses in the first-line complete cohort revealed stage-specific divergence. While ES-SCLC maintained robust discrimination in the validation set, LS-SCLC showed non-significant discrimination. This discrepancy likely reflects the reduced LS-SCLC validation sample size following the exclusion of discontinued patients (*n* = 34 in the first-line complete cohort vs. *n* = 39 in the complete-follow-up cohort), coupled with inherently lower event rates due to curative-intent therapy. The wide confidence intervals in the first-line complete cohort validation subgroup (spanning 1.0) further suggest an estimation uncertainty that precludes definitive conclusions regarding LS applicability. Thus, although the complete-follow-up cohort indicates potential utility in LS-SCLC, the first-line complete cohort showed inconsistent validation, and broad generalizability cannot be established. Given that ES-SCLC constitutes the majority of cases and has uniformly poor outcomes where accurate risk stratification is urgently needed, the model demonstrates clearer clinical utility in this population. Application to LS-SCLC requires cautious interpretation and further validation before routine implementation.

Several limitations warrant consideration. First, given the limited SCLC case number (*n* = 179) in the FinnGen GWAS, these results should be interpreted with caution, as low PP.H4 values may reflect insufficient statistical power rather than the biological absence of shared mechanisms. Second, the Elastic Net machine learning model demonstrated near-perfect discrimination (AUC = 0.996) in the validation cohort. Although this is concerning given the small training sample (*n* = 36), nested cross-validation, strict anti-leakage measures, and permutation testing (all *p* < 0.001) confirmed that the signal is not attributable to overfitting. The high AUC likely reflects genuine population heterogeneity. Nevertheless, prospective external validation is essential before clinical application. Third, the prognostic model was developed and internally validated at a single center in China, and its generalizability to other populations remains unknown. Fourth, limited SCLC resectability restricted tissue availability, necessitating the use of PBMCs for expression validation. The sample size for PBMC scRNA-seq (*n* = 3 per group) was exploratory and requires confirmation in larger cohorts. Fifth, some participants withdrew informed consent during follow-up. Ethical mandates required us to cease all further follow-up and remove their data completely from the analytical dataset. As a result, their clinical outcomes remain unknown, and we are unable to assess the potential impact of this data deletion on selection bias and the robustness of our survival estimates. Sixth, plasma IL18R1 was measured using ELISA, which requires standardization across institutions.

## 5. Conclusions

Integrative bioinformatics identified *ACE*, *AGER*, and *IL18R1* as candidate SCLC tumor suppressors, with machine learning validating a predictive signature driven by AGER and IL18R1. Single-cell profiling revealed predominant downregulation in immune and epithelial cells (dendritic cells, fibroblasts, T cells, and alveolar type II cells), potentially suppressing tumorigenesis via NLR/p53/Hh pathway modulation. A prognostic model incorporating plasma IL18R1 enabled robust risk stratification in ES-SCLC, supporting personalized management, though LS-SCLC applicability requires further validation.

## Data Availability

The publicly available datasets supporting this study are accessible via GEO (https://www.ncbi.nlm.nih.gov/geo/, accessed on 5 January 2022), GWAS Catalog (https://gwas.mrcieu.ac.uk/), and the eQTLGen consortium (https://eqtlgen.org/). Restrictions apply to the availability of raw PBMC transcriptomic data and patient clinical information due to privacy protection and ethical approval requirements. These data are available by request from the corresponding author. Aggregated data are provided in the Supplementary Materials.

## Supplementary Materials

The following supporting information can be downloaded at: https://www.mdpi.com/article/10.3390/cancers18101608/s1. Figure S1. WGCNA of the GSE149507 dataset and enrichment analyses; Figure S2. Regional association plots for Bayesian colocalization analysis of SMR-identified candidate genes with small cell lung cancer risk; Figure S3. Predicted probability distributions for five machine learning models stratified by outcome in the development and validation cohorts; Figure S4. Differential expression genes volcano plots (small cell lung cancer vs. normal lung) with top 5 hits labeled; Figure S5. Intercellular Communication Analysis; Figure S6. Gene Set Enrichment Analysis for *ACE*; Figure S7. Gene Set Enrichment Analysis for *AGER*; Figure S8. Gene Set Enrichment Analysis for *IL18R1*; Figure S9. Prognostic model performance in the complete case sensitivity analysis; Figure S10. Subgroup survival analysis by disease stage in the complete case sensitivity analysis. Table S1: Detailed information on the Blue Module in WGCNA; Table S2: Detailed information of DEGs; Table S3. Summary of 6 identified genes from the intersection of SMR, DEG, and WGCNA analyses; Table S4. Bayesian colocalization results for SMR-identified candidate genes with small cell lung cancer risk; Table S5. Baseline characteristics of the training and validation sets in the machine learning diagnostic model; Table S6: Performance indicator data of all the prediction models; Table S7: Baseline information for training and validation sets in complete-follow-up cohort; Table S8. Baseline information for training and validation sets in first-line complete cohort; Table S9. LASSO-penalized coefficients and multivariable Cox regression estimates for the 12 selected variables; Table S10. Detailed calibration metrics by deciles of predicted risk in the training and validation cohorts; Table S11. Subgroup analysis of PFS by disease stage in training and validation sets; Table S12. Subgroup survival analysis by disease stage in the complete-case sensitivity analysis.

## Abbreviations

The following abbreviations are used in this manuscript:

SCLC	Small Cell Lung Cancer
ES	Extensive-Stage
SMR	Summary-data-based Mendelian Randomization
WGCNA	Weighted Gene Co-expression Network Analysis
PBMCs	Peripheral Blood Mononuclear Cells
PFS	Progression-Free Survival
GWAS	Genome-Wide Association Study
GEO	Gene Expression Omnibus
eQTL	Expression Quantitative Trait Loci
cis-pQTLs	Cis-protein Quantitative Trait Loci
HEIDI	Heterogeneity in Dependent Instruments
FDR	False Discovery Rate
PPI	Protein-Protein Interaction
PP.H4	Posterior Probability of H4
DEGs	Differential Expression Genes
GS	Gene Significance
MM	Module Membership
AUC	Area Under the Curve
NCV	Nested Cross-Validation
GSEA	Gene Set Enrichment Analysis
LS	Limited-Stage
ULN	Upper Limit of Normal
ALT/AST	Alanine/Aspartate Aminotransferase
RECIST	Response Evaluation Criteria in Solid Tumors
BMI	Body Mass Index
RBC	Red Blood Cell
WBC	White Blood Cell
ProGRP	Pro-Gastrin-Releasing Peptide
NSE	Neuron-Specific Enolase
CEA	Carcinoembryonic Antigen
CYFRA21-1	Cytokeratin-19 Fragment
SCC	Squamous Cell Carcinoma Antigen
TT	Thrombin Time
PT	Prothrombin Time
APTT	Activated Partial Thromboplastin Time
LASSO	Least Absolute Shrinkage and Selection Operator
DCA	Decision Curve Analysis
CV	Cross-Validation
NLR	NOD-like Receptor
Hh	Hedgehog
pDC	Plasmacytoid Dendritic Cells
ROC	Receiver Operating Character
