# Supplementary material for: Integrative Multi-Omics Analysis Identifies IL18R1 as a Circulating Prognostic Biomarker for Risk Stratification in Extensive-Stage Small Cell Lung Cancer
Source: Cancers (Basel). 2026 May 15;18(10):1608. doi: 10.3390/cancers18101608 (PMC13204795; doi:10.3390/cancers18101608)
Supplement: Supplementary file 1 [file cancers-18-01608-s001.zip › Supplementary Figures.pdf]

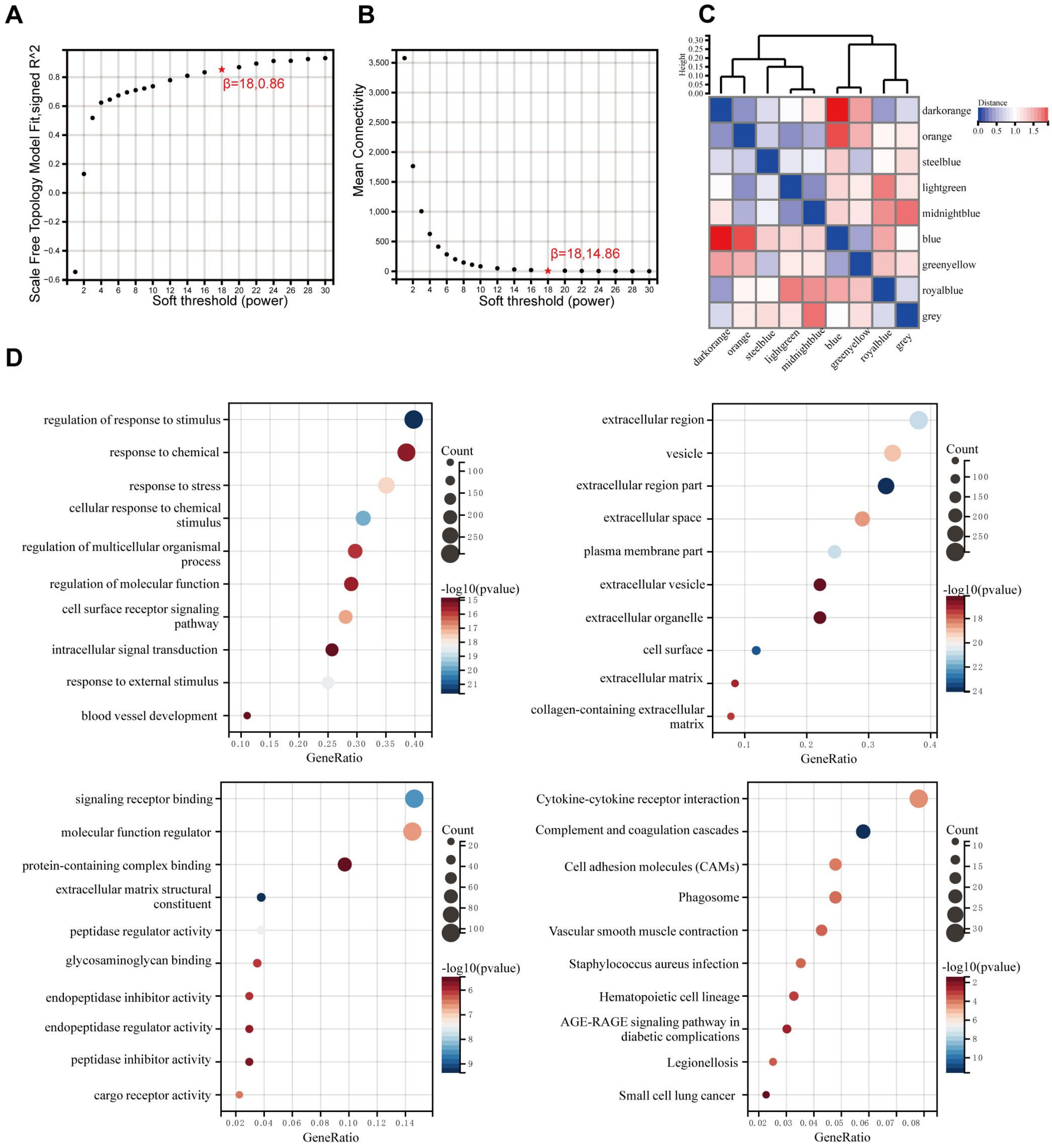

Figure S1.WGCNA Analysis of the GSE149507 Dataset and Enrichment Analyses.

(A) Scale-free topology analysis in weighted gene co-expression network (WGCNA). (B) Mean connectivity analysis in WGCNA. (C) Correlation between module eigengenes. (D) Functional enrichment analysis (GO biological processes and KEGG pathways) of hub genes identified at the intersection of WGCNA and DEG analyses.

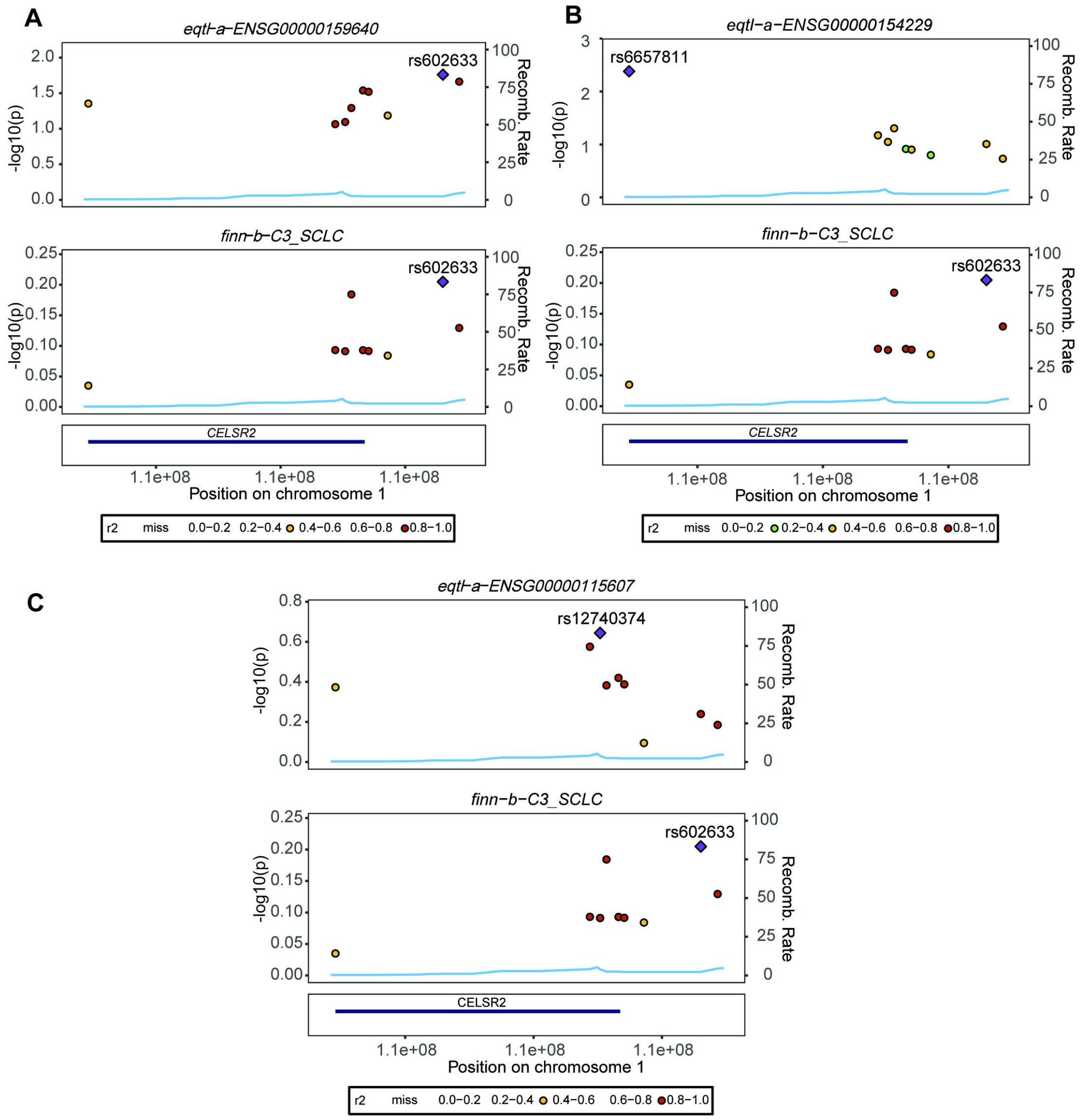

Figure S2. Regional association plots for Bayesian colocalization analysis of SMR-identified candidate genes with small cell lung cancer risk.

LocusZoom-style plots showing eQTL (upper panel) and SCLC GWAS (lower panel) association signals for (A) *ACE*, (B) *AGER*, and (C) *IL18R1*. Purple diamonds indicate lead SNPs. Colors represent linkage disequilibrium (r^2) with the lead SNP. Recombination rates are shown as light blue lines. All three genes are located within the linkage imbalance region of *CELSR2*.

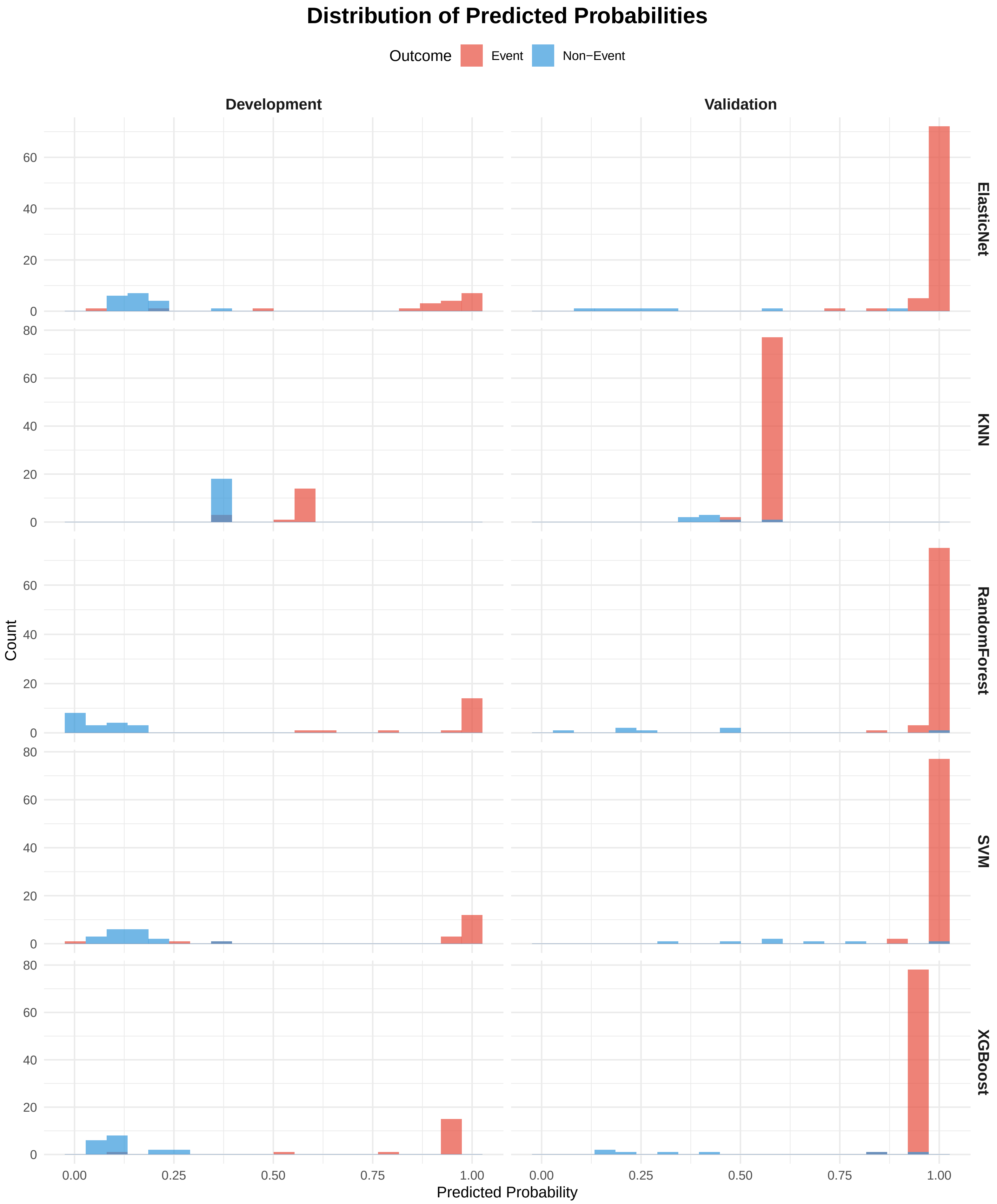

Figure S3. Predicted probability distributions for five machine learning models stratified by outcome in the development and validation cohorts.

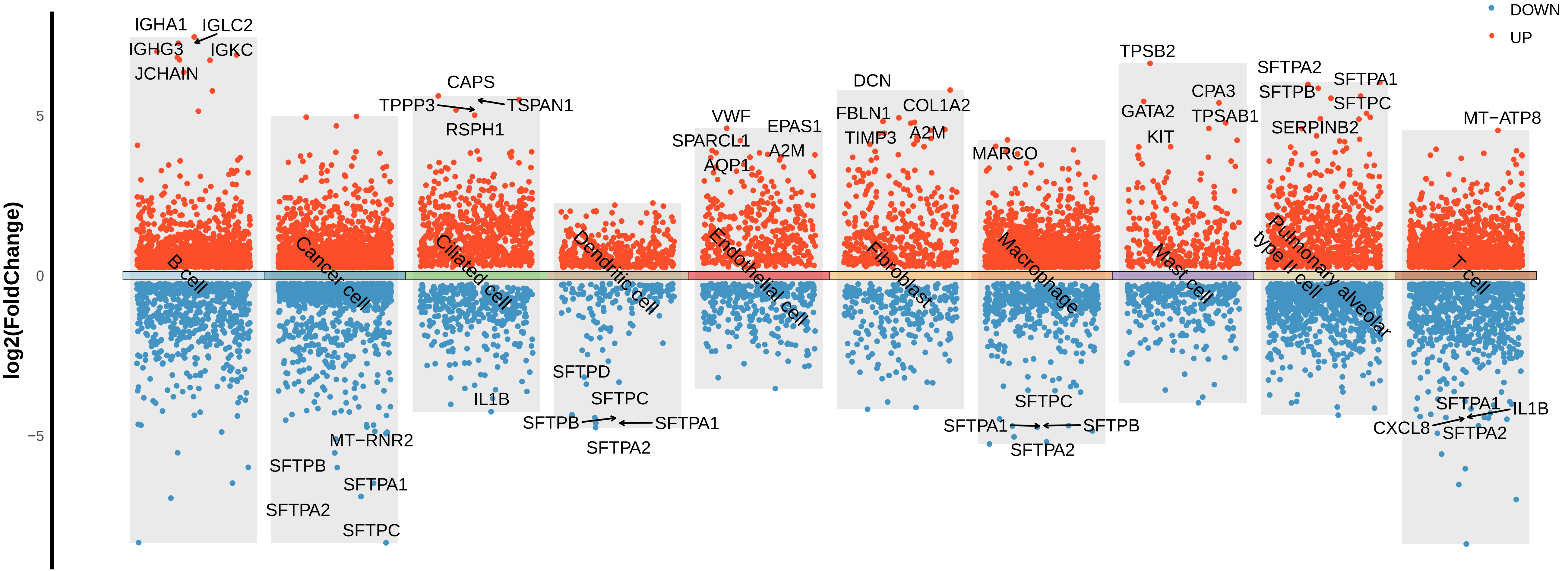

Figure S4. Differential expression genes volcano plots (small cell lung cancer vs. normal lung) with top 5 hits labeled.

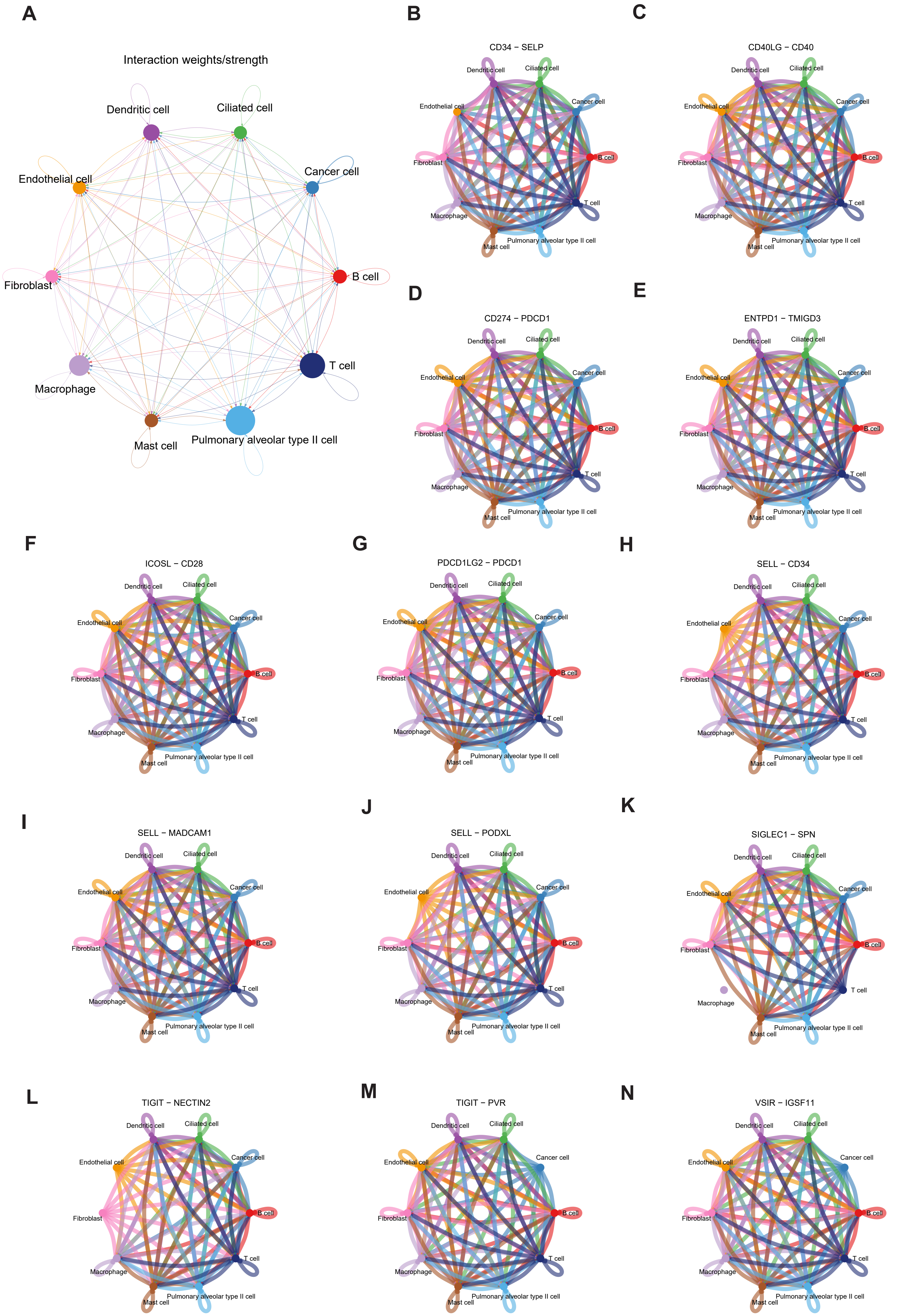
A
Interaction weights/strength
Dendritic cell
Ciliated cell
Cancer cell
Endothelial cell
Fibroblast
B cell
Macrophage
T cell
Mast cell
Pulmonary alveolar type II cell
B
CD34 – SELP
C
CD40LG – CD40
D
CD274 – PDCD1
E
ENTPD1 – TMIGD3
F
ICOSL – CD28
G
PDCD1LG2 – PDCD1
H
SELL – CD34
I
SELL – MADCAM1
J
SELL – PODXL
K
SIGLEC1 – SPN
L
TIGIT – NECTIN2
M
TIGIT – PVR
N
VSIR – IGSF11
Dendritic cell
Ciliated cell
Endothelial cell
Cancer cell
Fibroblast
B cell
Macrophage
T cell
Mast cell
Pulmonary alveolar type II cell

Figure S5. Intercellular Communication Analysis.

(A) Network diagram of interactions strength among 10 cell types. The arrows indicated signaling direction, and bigger circle meant higher strength of interactions. (B-N) Representative ligand-receptor pairs involved in communication between dendritic cells/fibroblasts and cancer cells.

GSEA result of *ACE*

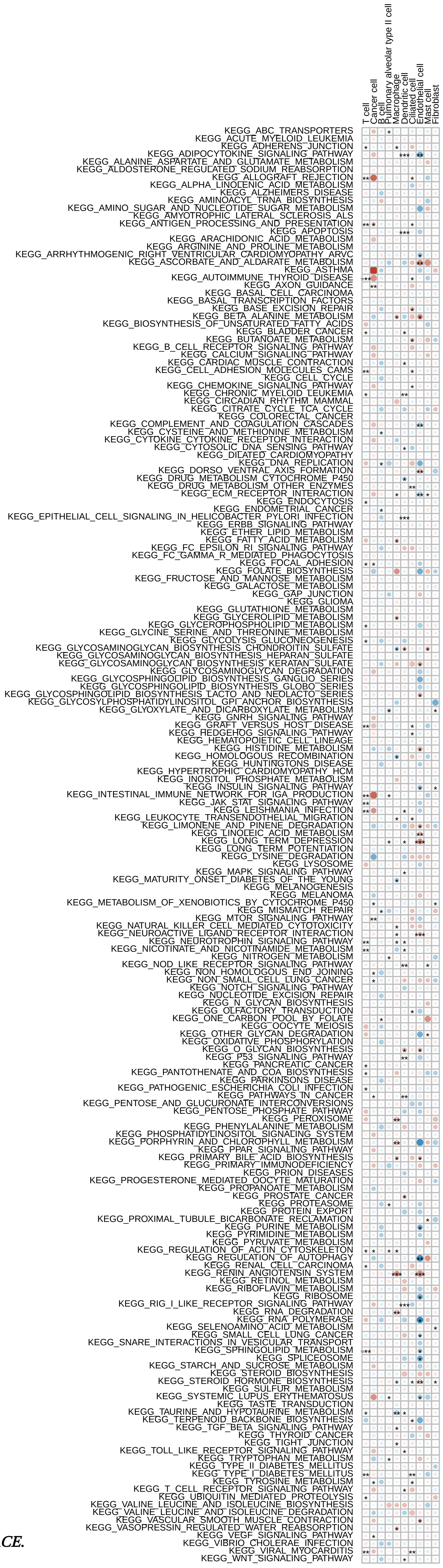

Figure S6. Gene Set Enrichment Analysis for *ACE*.

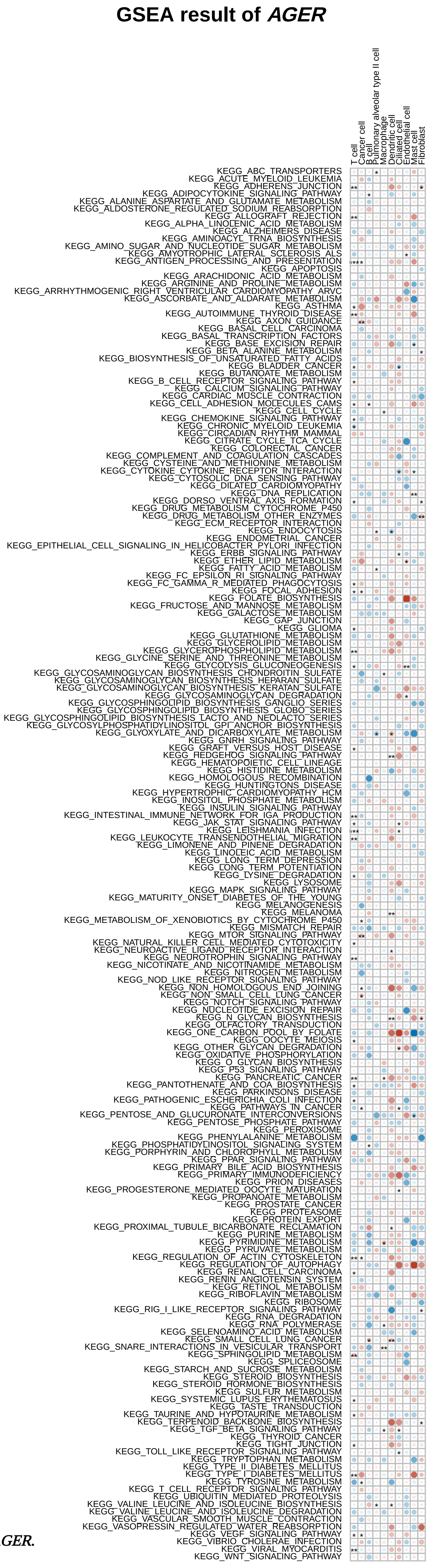

Figure S7. Gene Set Enrichment Analysis for *AGER*.

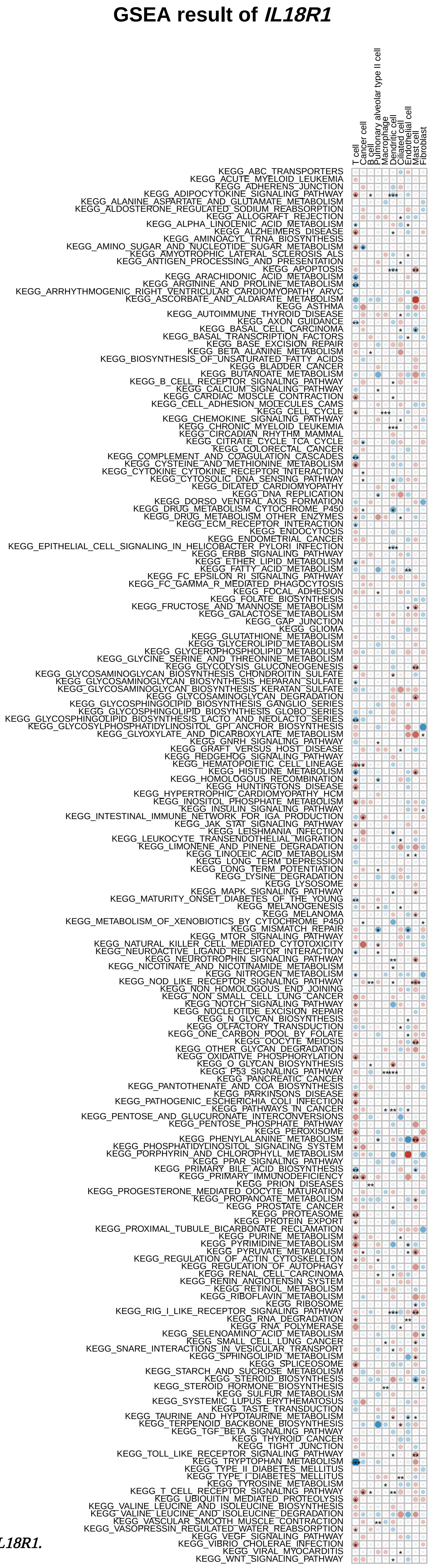

Figure S8. Gene Set Enrichment Analysis for *IL18R1*.

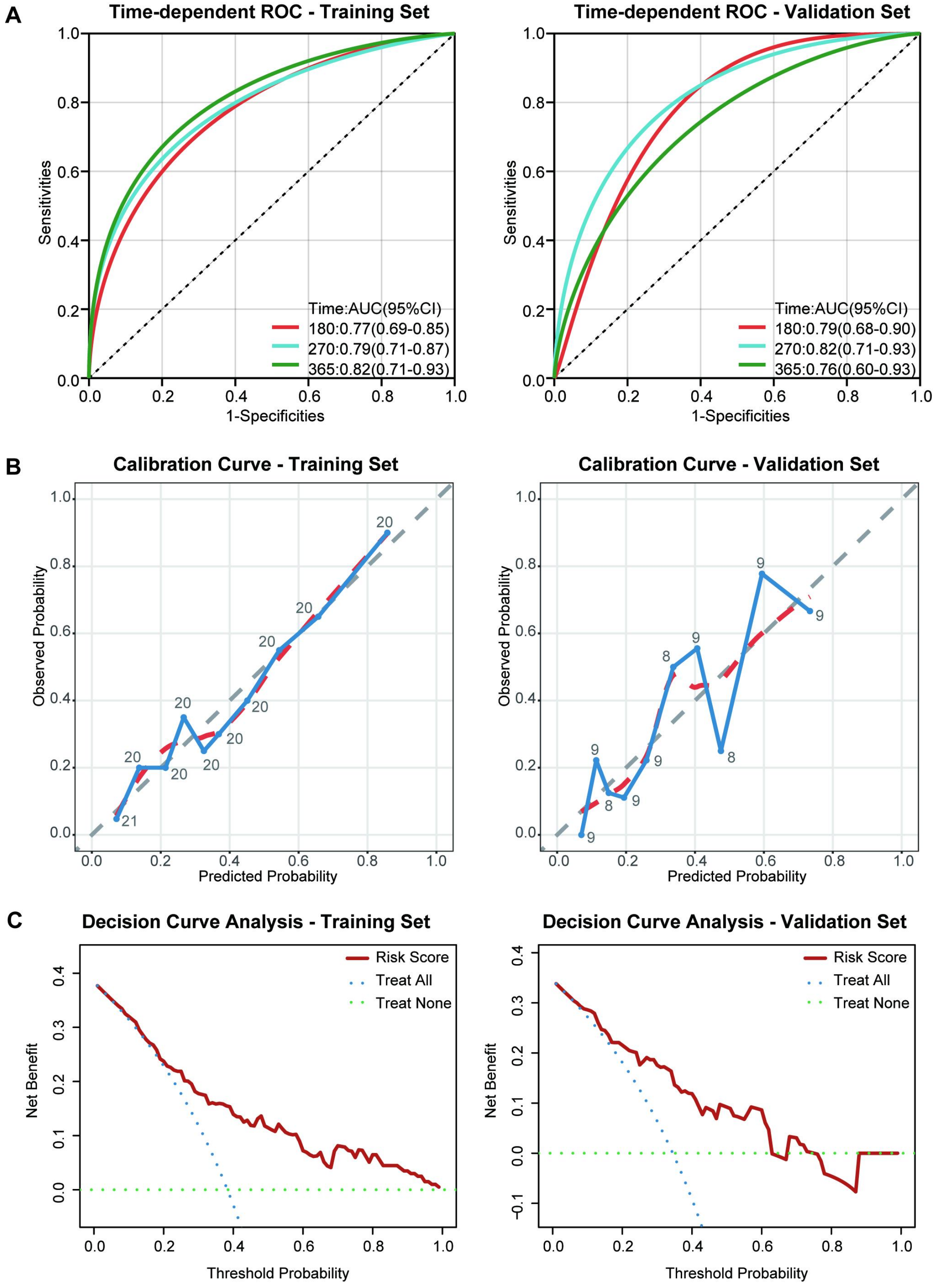

Figure S9. Prognostic model performance in the complete-case sensitivity analysis.

(A) Time-dependent receiver operating characteristic curves in the complete-case sensitivity analysis. AUC, area under the curve; CI, confidence interval. (B) Calibration plots in the complete-case sensitivity analysis. (C) Decision curve analysis in the complete-case sensitivity analysis.

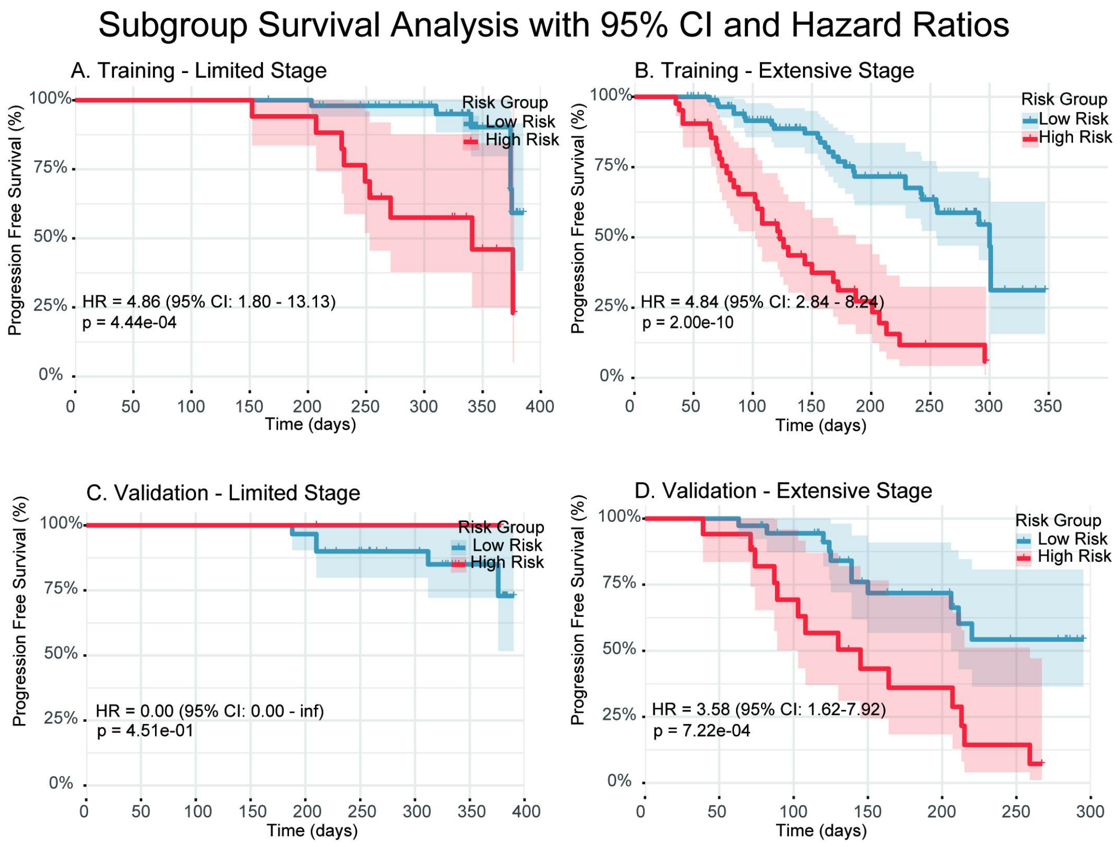

Figure S10. Subgroup survival analysis by disease stage in the complete-case sensitivity analysis.
